# Novel Pyrazine Analogs of Chalcones: Synthesis and Evaluation of Their Antifungal and Antimycobacterial Activity

**DOI:** 10.3390/molecules20011104

**Published:** 2015-01-12

**Authors:** Marta Kucerova-Chlupacova, Jiri Kunes, Vladimir Buchta, Marcela Vejsova, Veronika Opletalova

**Affiliations:** 1 Department of Pharmaceutical Chemistry and Pharmaceutical Analysis, Charles University in Prague, Faculty of Pharmacy in Hradec Kralove, Heyrovskeho 1203, 500 05 Hradec Kralove, Czech Republic; E-Mail: marta.kucerova@faf.cuni.cz; 2 Department of Inorganic and Organic Chemistry, Charles University in Prague, Faculty of Pharmacy in Hradec Kralove, Heyrovskeho 1203, 500 05 Hradec Kralove, Czech Republic; E-Mail: jiri.kunes@faf.cuni.cz; 3 Department of Clinical Microbiology, University Hospital Hradec Kralove, Sokolska 581, 500 05 Hradec Kralove, Czech Republic; E-Mails: vladimir.buchta@fnhk.cz (V.B.), marcela.vejsova@fnhk.cz (M.V.)

**Keywords:** tuberculosis, mycoses, chalcones, pyrazine derivatives

## Abstract

Infectious diseases, such as tuberculosis and invasive mycoses, represent serious health problems. As a part of our long-term efforts to find new agents for the treatment of these diseases, a new series of pyrazine analogs of chalcones bearing an isopropyl group in position 5 of the pyrazine ring was prepared. The structures of the compounds were corroborated by IR and NMR spectroscopy and their purity confirmed by elemental analysis. The susceptibility of eight fungal strains to the studied compounds was tested. The results have been compared with the activity of some previously reported propyl derivatives. The only strain that was susceptible to the studied compounds was *Trichophyton mentagrophytes*. It was found that replacing a non-branched propyl with a branched isopropyl did not have a decisive and unequivocal influence on the* in vitro* antifungal activity against *T. mentagrophytes*. *In vitro* activity against *Trichophyton mentagrophytes* comparable with that of fluconazole was exhibited by nitro-substituted derivatives. Unfortunately, no compound exhibited efficacy comparable with that of terbinafine, which is the most widely used agent for treating mycoses caused by dermatophytes. Some of the prepared compounds were assayed for antimycobacterial activity against *M. tuberculosis* H_37_Rv. The highest potency was also displayed by nitro-substituted compounds. The results of the present study are in a good agreement with our previous findings and confirm the positive influence of electron-withdrawing groups on the B-ring of chalcones on the antifungal and antimycobacterial activity of these compounds.

## 1. Introduction

Infectious diseases used to be, and in some regions of the world still are, the major cause of death. Tuberculosis remains a severe global public health threat, especially in the context of the emergence of multidrug-resistant (MDR) and extensively drug resistant (XDR) strains in all countries of the world [1,2,3,4,5,6]. Fungal infections are a global challenge as well. Candidiasis is one of the most frequent fungal diseases. The epidemiology of mycoses has changed over the last two decades. Whilst the incidence of *Candida albicans* was decreased in many countries, the proportion of species other than *C. albicans* was increased, particularly at intensive care units [7]. Apart from *Candida*, other serious fungal pathogens participate in an increased morbidity and mortality in immunocompromised patients. *Aspergillus* species Mucorales represent a leading etiology of invasive mycoses, especially in connection with risk and predisposing factors such as transplantation, immunosuppressive therapy, catheterization, poorly controlled diabetes, iron overload and major trauma [8,9,10].

Chalcones are natural products that are not only important intermediates for the biosynthesis of other flavonoids but exhibit a variety of biological effects by themselves [11]. The chalcone 1,3-diphenylprop-2-en-1-one skeleton is a privileged structure in drug design [12,13,14], and many synthetic chalcones and their heterocyclic congeners have been studied. Several reviews dealing with the preparation, properties and biological activities of these compounds have been published recently [15,16,17,18], and various studies dealing with the biological activities of heterocyclic analogs of chalcones have appeared [19,20,21,22,23].

Studies of the antimicrobial properties of various pyrazine derivatives have a long tradition at the Faculty of Pharmacy in Hradec Kralove and at the Department of Clinical Microbiology of the University Hospital Hradec Kralove [24,25,26,27,28,29,30,31]. The present paper is a continuation of our earlier work. In our previous papers [32,33,34] synthesis and* in vitro* antifungal and antimycobacterial activity of (*E*)-1-(5-alkylpyrazin-2-yl)-3-(subst. phenyl)prop-2-en-1-ones, where alkyl is butyl, isobutyl, *tert*-butyl or propyl, were reported. Derivatives without alkyl moieties on the pyrazine ring were included in these studies as well. Nonetheless, the influence of alkyl substitution on antimicrobial potency could not be clearly determined. In a series of (*E*)-1-(5-alkylpyrazin-2-yl)-3-(hydroxyphenyl)prop-2-en-1-ones derivatives with hydrogen or a non-branched alkyl on the pyrazine ring exhibited the highest antifungal potency, whilst substitution with *tert*-butyl seemed to be favorable for antimycobacterial potency [32]. A similar trend was later observed with (*E*)-1-(5-alkylpyrazin-2-yl)-3-(nitrophenyl)prop-2-en-1-ones [34]. Therefore we decided to prepare a series of (*E*)-1-(5-isopropylpyrazin-2-yl)-3-(subst. phenyl)prop-2-en-1-ones and compare their* in vitro* antifungal and antimycobacterial activities with those displayed previously by analogous propyl derivatives.

## 2. Results and Discussion

### 2.1. Chemistry

**Scheme 1:**
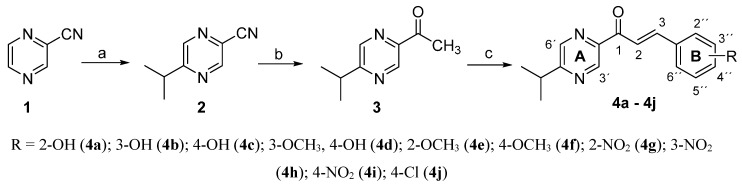
Synthesis of the compounds **4a**–**4j**. *Reagents and conditions:* (a) isobutyric acid, AgNO_3_, (NH_4_)_2_S_2_O_8_, water, 80 °C; (b) CH_3_MgI, Et_2_O; (c) substituted benzaldehyde, pyridine, Et_2_NH.

The studied compounds were prepared using the method described in our previous papers [32,33,34]. Pyrazine-2-carbonitrile (**1**) was submitted to Minisci radical alkylation [35,36,37] to yield 5-isopropylpyrazine-2-carbonitrile (**2**). Although the Minisci reaction has been widely used in synthetic organic chemistry [38] it was first applied to homolytic alkylation of pyrazine-2-carbonitrile by our research group [39,40]. 5-Isopropylpyrazine-2-carbonitrile (**2**) was then converted to 1-(5-isopropylpyrazin-2-yl)ethan-1-one (**3**) using a procedure described in our previous papers [39,40]. Modified Claisen-Schmidt condensation of **3** with substituted benzaldehydes gave the pyrazine congeners of chalcones **4a**–**4j** (Scheme 1). To separate the products from the reaction mixtures, column chromatography using a mixture of light petroleum-ethyl acetate was necessary (except for compound **4h**). The yields of the products ranged between 18%–43% for most examples. However, isolation of compounds **4e**, **4f** and **4j** was difficult. A mobile phase with a low content of ethyl acetate (90:10) was used, but the yields remained very low (<10%). A similar problem has previously been observed with (*E*)-1-(5-alkylpyrazin-2-yl)-3-(2-methoxyphenyl)prop-2-en-1-ones, (*E*)-1-(5-alkylpyrazin-2-yl)-3-(4-methoxyphenyl)prop-2-en-1-ones and (*E*)-1-(5-alkylpyrazin-2-yl)-3-(4-chlorophenyl)prop-2-en-1-ones where alkyl was propyl, butyl, isobutyl or *tert*-butyl [33,41]. This may be due to the lower reactivity of methoxy- and chloro-substituted benzaldehydes in the Claisen-Schmidt reaction or the high lipophilicity (log *P* = 2.8 for **4e** and **4f** and 3.48 for **4j**) of these compounds which complicates their chromatographic separation. Purity of the products was checked by elemental analysis. Their structures were confirmed by their IR and NMR spectra. The values of the spin interaction constant *J* (15–16 Hz) corresponds to an *E*‑configuration on the double bond.

### 2.2. Biological Evaluation

#### 2.2.1. Antifungal Activity

Like the chalcone analogs previously prepared by us [32,33,34], the (*E*)-1-(5-isopropylpyrazin-2-yl)-3-(substituted phenyl)-prop-2-en-1-ones reported here were tested as potential antimycotic and antituberculous drugs. *In vitro* susceptibility of eight fungal strains to the studied compounds was determined and results are summarized in Table 1. MICs of previously reported propyl derivatives **5a**–**5j** are given for comparison. The compounds were inactive or only weekly active against most strains, hence some comparison is possible only for *Trichophyton mentagrophytes.* In five cases (**4a** and **5a**, **4b** and **5b**, **4c** and **5c**, **4d** and **5d**, **4j** and **5j**) the propyl derivative exhibited better potency than the corresponding isopropyl congener, which is in agreement with trends indicated in the Introduction. However, in four cases (**4e** and **5e**, **4f** and **5f**, **4g** and **5g**, **4i** and **5i**) an opposite trend was observed, and in case of **4h** and **5h** comparison is not possible due to the insolubility of compound **4h**.

Moreover, the differences in the potencies of propyl and isopropyl derivatives are sometimes very subtle. Therefore, it can only be concluded that replacing a non-branched propyl with a branched isopropyl does not have a decisive and unequivocal influence on the* in vitro* antifungal activity against *T. mentagrophytes*. Potency of (*E*)-1-(5-isopropylpyrazin-2-yl)-3-(2-nitrophenyl)-prop-2-en-1-one (**4g**), and (*E*)-1-(5-isopropyl-pyrazin-2-yl)-3-(4-nitrophenyl)prop-2-en-1-one (**4i**) was comparable to that of fluconazole, which is sometimes used for the treatment of mycoses caused by *Trichophyton* spp. [42,43], but lower than that of voriconazole and terbinafine. Terbinafine is most widely used agent to treat mycoses caused by dermatophytes and other fungi [42,44]. Voriconazole belongs to the highly effective systemic antifungal drugs with a favourable risk-benefit ratio, and with distinct* in vitro* activity against dermatophytes, yeasts and some molds [45] but clinically it is used to treat invasive aspergillosis [46,47]. The good potency of the nitro-substituted derivatives **4g** and **4i** is in a good agreement with our previous results [34], confirming the positive influence of a nitro group on the B‑ring on the antifungal potency of chalcone-like derivatives.

**Table 1:** Antifungal activity of isopropyl derivatives **4a**–**4j** and propyl derivatives **5a**–**5j** (compared to fluconazole, voriconazole and terbinafine).

Compd.	MIC (μmol/L) IC_80_ or greater for yeasts and yeast-like organisms IC_50_ or greater for molds							
	CA	CT	CK	CG	TA	AF	LC	TM
	*24 h*	*24 h*	*24 h*	*24 h*	*24 h*	*24 h*	*24 h*	*72 h*
	*48 h*	*48 h*	*48 h*	*48 h*	*48 h*	*48 h*	*48 h*	*120 h*
**4a**	˃125	˃125	˃125	˃125	˃125	˃125	˃125	˃125
	˃125	˃125	˃125	˃125	˃125	˃125	˃125	˃125
**5a**	˃500	˃500	˃500	˃500	˃500	500	500	7.81
	˃500	˃500	˃500	˃500	˃500	˃500	˃500	7.81
**4b**	62.5	125	˃250	125	125	62.5	125	15.62
	125	˃250	˃250	˃250	˃250	˃250	˃250	31.25
**5b**	62.5	62.5	˃125	62.5	62.5	31.25	˃125	15.62
	62.5	125	˃125	62.5	˃125	˃125	˃125	15.62
**4c**	62.5	˃250	˃250	˃250	˃250	62.5	˃250	62.5
	˃250	˃250	˃250	˃250	˃250	250	˃250	125
**5c**	˃500	˃500	˃500	˃500	˃500	˃500	˃500	31.25
	˃500	˃500	˃500	˃500	˃500	˃500	˃500	62.5
**4d**	˃250	˃250	˃250	˃250	˃250	˃250	˃250	˃250
	˃250	˃250	˃250	˃250	˃250	˃250	˃250	˃250
**5d**	˃125	˃125	˃125	˃125	˃125	˃125	˃125	˃125
	˃125	˃125	˃125	˃125	˃125	˃125	˃125	ND ^a^
**4e**	˃125	˃125	˃125	˃125	˃125	˃125	˃125	31.25
	˃125	˃125	˃125	˃125	˃125	˃125	˃125	62.5
**5e**	500	˃500	˃500	˃500	˃500	500	˃500	500
	˃500	˃500	˃500	˃500	˃500	˃500	˃500	500
**4f**	250	˃500	250	˃500	˃500	˃500	˃500	125
	250	˃500	250	˃500	˃500	˃500	˃500	125
**5f**	˃125	˃125	˃125	˃125	˃125	˃125	˃125	˃125
	˃125	˃125	˃125	˃125	˃125	˃125	˃125	˃125
**4g**	32.15	˃250	˃250	˃250	˃250	125	125	**7.81**
	125	˃250	˃250	˃250	˃250	˃250	˃250	**7.81**
**5g**	˃500	˃500	˃500	˃500	˃500	˃500	˃500	250
	˃500	˃500	˃500	˃500	˃500	˃500	˃500	250
**4h**	NS ^b^	NS ^b^	NS ^b^	NS ^b^	NS ^b^	NS ^b^	NS ^b^	NS ^b^
	NS ^b^	NS ^b^	NS ^b^	NS ^b^	NS ^b^	NS ^b^	NS ^b^	NS ^b^
**5h**	15.62	62.5	32.15	125	˃250	62.5	˃250	7.81
	62.5	˃250	˃250	˃250	˃250	˃250	˃250	15.62
**4i**	7.81	˃250	˃250	˃250	˃250	125	250	**3.90**
	62.5	˃250	˃250	˃250	˃250	˃250	250	**7.81**
**5i**	˃62.5	˃62.5	˃62.5	˃62.5	˃62.5	˃62.5	˃62.5	7.81
	˃62.5	˃62.5	˃62.5	˃62.5	˃62.5	˃62.5	˃62.5	15.62
**4j**	˃125	˃125	˃125	˃125	˃125	˃125	˃125	≤62.5
	˃125	˃125	˃125	˃125	˃125	˃125	˃125	≤62.5
**5j**	>62.5	>62.5	>62.5	>62.5	>62.5	>62.5	>62.5	7.81
	>62.5	>62.5	>62.5	>62.5	>62.5	>62.5	>62.5	7.81
**FLU**	0.24	˃500	125	41.64	250	˃500	˃500	6.51
	0.24	˃500	250	250	500	˃500	˃500	104
**VOR**	0.005	125	0.65	83.58	3.26	0.49	208	0.08
	0.007	250	1.95	250	14.32	1.3	250	0.12
**TER**	˃6.86 ^c^	˃6.86 ^c^	˃6.86 ^c^	˃6.86 ^c^	NA ^d^	NA ^d^	NA ^d^	0.01–1.72 ^c^

Notes: **CA*** = Candida albicans* ATCC 44859, **CT** = *Candida tropicalis* 156, **CK** = *Candida krusei* E 28, **CG** = *Candida glabrata* 20/I, **TA** = *Trichosporon asahii* 1188, **AF** = *Aspergillus fumigatus* 231, **LC** = *Lichtheimia corymbifera* (formerly *Absidia corymbifera* [48]) 272, **TM** = *Trichophyton mentagrophytes* 445; **FLU** = fluconazole, **VOR** = voriconazole, **TER** = terbinafine; ^a^: not determined, ^b^: not soluble, ^c^: IC_50_ after 7 days of incubation [49], ^d^: not available.

#### 2.2.2. Antimycobacterial Activity

Selected compounds were submitted to evaluation of antimycobacterial activity in the Tuberculosis Antimicrobial Acquisition and Coordination Facility (TAACF) through a research and development contract with the U.S. National Institute of Allergy and Infectious Diseases. The results are shown in Table 2. As expected according to our previous results [34], the best inhibition was displayed by 2-nitro (**4g**) and 4-nitro (**4i**) derivatives, but they were less potent than the previously reported (*E*)-1-(5-*tert*-butylpyrazin-2-yl)-3-(4-nitrophenyl)prop-2-en-1-one (MIC_90_ = 0.78 μg/mL). This confirms that *tert*-butyl is the best substituent for antimycobacterial potency. A lower efficacy was observed with 2-hydroxy- (**4a**) and 4-hydroxy- (**4c**) substituted compounds, which is also in agreement with our previous results [32,34]. 

**Table 2:** Antimycobacterial activity of compounds **4a**–**4j** and **5a**–**5j** compared to isoniazid and rifampicin.

Compd.	R	% Inhibition at 6.25 μg/mL	MIC_90_ (μg/mL)	CC_50_ (μg/mL)	SI
**4a**	2-OH	76	ND	ND	ND
**5a**	2-OH	50	ND	ND	ND
**4b**	3-OH	0	ND	ND	ND
**5b**	3-OH	0	ND	ND	ND
**4c**	4-OH	59	ND	ND	ND
**5c**	4-OH	35	ND	ND	ND
**4d**	3-OCH_3_, 4-OH	ND	ND	ND	ND
**5d**	3-OCH_3_, 4-OH	20	ND	ND	ND
**4e**	2-OCH_3_	ND	ND	ND	ND
**5e**	2-OCH_3_	71	ND	ND	ND
**4f**	4-OCH_3_	ND	ND	ND	ND
**5f**	4-OCH_3_	ND	ND	ND	ND
**4g**	2-NO_2_	**97**	**6.25**	**0.84**	**0.13**
**5g**	2-NO_2_	57	ND	ND	ND
**4h**	3-NO_2_	0	ND	ND	ND
**5h**	3-NO_2_	0	ND	ND	ND
**4i**	4-NO_2_	**91**	**6.25**	**1.14**	**0.18**
**5i**	4-NO_2_	100	>6,25	ND	ND
**4j**	4-Cl	0	ND	ND	ND
**5j**	4-Cl	12	ND	ND	ND
**isoniazid**		ND	0.025–0.05	>1000	>40,000
**rifampicin**		98	0.015–0.125	>100	>800

Note: ND = not determined.

For moving compounds into* in vivo* testing MIC ≤ 6.25 µg/mL and an selectivity index (the ratio of the measured CC_50_ in VERO cells to the MIC) SI ≥ 10 are required. Unfortunately, the selectivity indexes of the two promising compounds were too low.

## 3. Experimental Section

### 3.1. Chemistry

#### 3.1.1. Materials and Methods

Pyrazine-2-carbonitrile (Sigma-Aldrich, Prague, Czech Republic) was used as a starting compound. 5-Isopropylpyrazine-2-carbonitrile and 1-(5-isopropylpyrazin-2-yl)ethan-1-one were prepared as described previously [40]. Commercially available substituted benzaldehydes (Sigma-Aldrich) were used as the starting materials. Silpearl (Kavalier, Votice, Czech Republic) was used for flash column chromatography. The purity of the products was checked by TLC on Silufol UV 254 plates (Kavalier). Mixtures of light petroleum and ethyl acetate were used for TLC. Analytical samples were dried over anhydrous phosphorus pentoxide under reduced pressure at room temperature. Melting points were determined on a Boëtius apparatus and are uncorrected. Elemental analyses were performed on an EA 1110 CHNS instrument (CE Instruments, Milano, Italy). Infrared spectra were recorded in KBr pellets on a Nicolet Impact 400 IR spectrophotometer (Thermo Scientific, Waltham, MA, USA). Characteristic wavenumbers are given in cm^−1^. ^1^H- and ^13^C-NMR spectra were recorded at ambient temperature on a Varian Mercury-Vx BB 300 spectrometer (Varian Corp., Palo Alto, CA, USA) operating at 300 MHz for ^1^H and 75 MHz for ^13^C. Chemical shifts were recorded as δ values in ppm, and were indirectly referenced to tetramethylsilane (TMS) via the solvent signal (2.49 for ^1^H, 39.7 for ^13^C in DMSO-*d*_6_ and 7.26 for ^1^H, 77.0 for ^13^C in CDCl_3_). Coupling constants *J* are given in Hz.

#### 3.1.2. Synthesis of (*E*)-1-(5-isopropylpyrazin-2-yl)-3-phenylprop-2-en-1-ones **4a**–**4j**

1-(5-Isopropylpyrazin-2-yl)ethan-1-one (0.01 mol) and a substituted benzaldehyde (0.01 mol) were dissolved in pyridine (4.4 mL). Diethylamine (0.73 g, 0.01 mol) was added, and the reaction mixture was stirred at 80–120 °C for 2 h. After cooling, the mixture was poured into ice water (200 mL), acidified to pH 3 with a few drops of acetic acid, and then refrigerated for 24 h. The separation of crude products from water depended on their character. Solid product **4h** was filtered off and crystallized from anhydrous ethanol. Oily mixtures were extracted with diethyl ether and subjected to flash chromatography on silica gel. Light petroleum–ethyl acetate 60:40 (v/v) was used as the eluent for compounds **4a**–**4d** and **4g**, 80:20 (v/v) ratio of the two solvents was used for **4i**, and compounds **4e**, **4f** and **4j** were separated using light petroleum-ethyl acetate 90:10 (v/v). The fractions containing the desired compounds were combined and crystallized from anhydrous ethanol. Using this procedure, the following compounds were obtained:

*(E)-3-(2-Hydroxyphenyl)-1-(5-isopropylpyrazin-2-yl)prop-2-en-1-one* (**4a**). Yellow solid; yield 30%; m.p. 175–178 °C; IR: 1652 (C=O), 1584 (C=C); ^1^H-NMR (DMSO-*d*_6_): 10.41 (s, 1H, OH), 9.14 (d, 1H, *J* = 1.4 Hz, H-3'), 8.77 (d, 1H, *J* = 1.4 Hz, H-6'), 8.20 (d, 1H, *J* = 16.2 Hz, H-3), 8.09 (d, 1H, *J* = 16.2 Hz, H-2), , 6.98–6.92 (m, 1H, *J* = 1.9 Hz, H-3''), 7.69 (dd, 1H, *J* = 1.4 and 7.7 Hz, H-6''), 7.32–7.25 (m, 1H, H-4''), 6.91–6.84 (m, 1H, H-5''), 3.30–3.15 (m, 1H, CH), 1.29 (d, 6H, *J* = 7.1 Hz, CH_3_); ^13^C-NMR (DMSO-*d*_6_): 188.5, 165.4, 157.9, 146.4, 143.2, 142.4, 140.7, 132.6, 129.7, 121.4, 120.1, 119.8, 116.6, 33.7, 22.0; EA for C_16_H_16_N_2_O_2_ (268.32) calculated 71.62% C, 6.01% H, 10.44% N, found 71.53% C, 6.15% H, 10.37% N.

*(E)-3-(3-Hydroxyphenyl)-1-(5-isopropylpyrazin-2-yl)prop-2-en-1-one* (**4b**). Yellow solid; yield 18%; m.p. 154–157 °C; IR: 1637 (C=O), 1608 (C=C); ^1^H-NMR (DMSO-*d*_6_): 9.69 (bs, 1H, OH), 9.15 (d, 1H, *J* = 1.4 Hz, H-3'), 8.77 (d, 1H, *J* = 1.4 Hz, H-6'), 8.05 (d, 1H, *J* = 16.2 Hz, H-3), 7.77 (d, 1H, *J* = 16.2 Hz, H-2), 7.69 (dd, 1H, *J* = 1.4 and 7.7 Hz, H-6''), 7.31–7.15 (m, 3H, H-2'', H-5'', H-6''), 6.91–6.85 (m, 1H, H-4''), 3.32–3.15 (m, 1H, CH), 1.29 (d, 6H, *J* = 6.9 Hz, CH_3_); ^13^C-NMR (DMSO-*d*_6_): 188.1, 165.6, 158.0, 146.1, 144.9, 143.3, 142.5, 135.8, 130.4, 120.4, 120.4, 118.6, 114.8, 33.7, 22.0; EA for C_16_H_16_N_2_O_2_ (268.32) calculated 71.62% C, 6.01% H, 10.44% N, found 71.53% C, 6.15% H, 10.37% N.

*(E)-3-(4-Hydroxyphenyl)-1-(5-isopropylpyrazin-2-yl)prop-2-en-1-one* (**4c**). Yellow solid; yield 25%; m.p. 155–158 °C; IR: 1665 (C=O), 1592 (C=C); ^1^H-NMR (CDCl_3_): 9.28 (d, 1H, *J* = 1.4 Hz, H-3'), 8.57 (d, 1H, *J* = 1.4 Hz, H-6'), 8.04 (d, 1H, *J* = 15.9 Hz, H-3), 7.92 (d, 1H, *J* = 15.9 Hz, H-2), 7.66–7.60 (m, 2H, AA'BB', H-2'', H-6''), 6.96–6.86 (m, 2H, AA'BB', H-3'', H-5''), 6.11 (s, 1H, OH), 3.31–3.16 (m, 1H, CH), 1.39 (d, 6H, *J* = 7.1 Hz, CH_3_); ^13^C-NMR (CDCl_3_): 188.6, 165.5, 158.5, 146.6, 145.4, 143.9, 141.5, 131.1, 127.7, 117.9, 116.0, 34.4, 22.0; EA for C_16_H_16_N_2_O_2_ (268.32) calculated 71.62% C, 6.01% H, 10.44% N, found 71.88% C, 6.01% H, 10.55% N.

*(E)-3-(4-Hydroxy-3-methoxyphenyl)-1-(5-isopropylpyrazin-2-yl)prop-2-en-1-one* (**4d**). Orange-yellow solid; yield 38%; m.p. 175–177 °C; IR: 1662 (C=O), 1611 (C=C); ^1^H-NMR (CDCl_3_): 9.28 (d, 1H, *J* = 1.4 Hz, H-3'), 8.56 (d, 1H, *J* = 1.4 Hz, H-6'), 8.01 (d, 1H, *J* = 15.9 Hz, H-3), 7.91 (d, 1H, *J* = 15.9 Hz, H-2), 7.28 (dd, 1H, J = 1.9 and 8.2, H-6'', 7.22 (d, 1H, *J* = 1.9, H-2''), 6.96 d, 1H, *J* = 8.2, H-5''), 6.01 (s, 1H, OH), 3.98 (s, 3H, OCH_3_), 3.29–3.16 (m, 1H, CH), 1.38 (d, 6H, *J* = 6.8 Hz, CH_3_); ^13^C-NMR (CDCl_3_): 188.4, 165.5, 148.6, 146.8, 146.6, 145.7, 144.0, 141.4, 127.5, 124.4, 117.8, 114.8, 110.0, 56.0, 34.3, 22.0; EA for C_17_H_18_N_2_O_3_ (298.34) calculated 68.44% C, 6.08% H, 9.39% N, found 68.67% C, 6.29% H, 9.23% N.

*(E)-1-(5-Isopropylpyrazin-2-yl)-3-(2-methoxyphenyl)prop-2-en-1-one* (**4e**). Yellow solid; yield 3%; m.p. 59–63 °C; IR: 1668 (C=O), 1592 (C=C); ^1^H-NMR (CDCl_3_): 9.27 (d, 1H, *J* = 1.4 Hz, H-3'), 8.56 (d, 1H, *J* = 1.4 Hz, H-6'), 8.34 (d, 1H, *J* = 16.2 Hz, H-3), 8.18 (d, 1H, *J* = 16.2 Hz, H-2), 7.69 (dd, 1H, *J* = 1.7 and 7.7 Hz, H-6''), 7.43–7.35 (m, 1H, H-4''), 7.03–6.92 (m, 1H, H-3''and H-5''), 3.92 (s, 3H, OCH_3_), 3.30–3.14 (m, 1H, CH), 1.38 (d, 6H, *J* = 6.9 Hz, CH_3_); ^13^C-NMR (CDCl_3_): 188.9, 165.3, 159.0, 146.7, 143.9, 141.5, 140.5, 132.1, 129.0, 123.9, 120.7, 111.2, 55.5, 34.3, 22.0; EA for C_17_H_18_N_2_O_2_ (282.34) calculated 72.32% C, 6.43% H, 9.92% N, found 72.33% C, 6.64% H, 10.00% N.

*(E)-1-(5-Isopropylpyrazin-2-yl)-3-(4-methoxyphenyl)prop-2-en-1-one* (**4f**). Yellow solid; yield 2%; m.p. 100–101 °C; IR: 1662 (C=O), 1583 (C=C); ^1^H-NMR (CDCl_3_): 9.27 (d, 1H, *J* = 1.4 Hz, H-3'), 8.55 (d, 1H, *J* = 1.4 Hz, H-6'), 8.05 (d, 1H, *J* = 15.9 Hz, H-3), 7.93 (d, 1H, *J* = 15.9 Hz, H-2), 7.72–7.64 (m, AA'BB', 2H, H-2'', H-6''), 6.98–6.88 (m, AA'BB', 2H, H-3'', H-5''), 3.86 (s, 3H, OCH_3_), 3.31–3.12 (m, 1H, CH), 1.38 (d, 6H, *J* = 6.9 Hz, CH_3_); ^13^C-NMR (CDCl_3_): 188.5, 165.4, 161.9, 146.6, 145.1, 143.9, 141.5, 130.7, 127.7, 118.0, 114.4, 55.4, 34.3, 22.0; EA for C_17_H_18_N_2_O_2_ (282.34) calculated 72.32% C, 6.43% H, 9.92% N, found 72.07% C, 6.71% H, 10.01% N.

*(E)-1-(5-Isopropylpyrazin-2-yl)-3-(2-nitrophenyl)prop-2-en-1-one* (**4g**). Yellow solid; yield 21%; m.p. 101–104 °C; IR: 1672 (C=O), 16.04 (C=C); ^1^H-NMR (CDCl_3_): 9.28 (d, 1H, *J* = 1.4 Hz, H-3'), 8.55 (d, 1H, *J* = 1.4 Hz, H-6'), 8.35 (d, 1H, *J* = 15.9 Hz, H-3), 8.05 (d, 1H, *J* = 15.9 Hz, H-2), 8.08–8.03 (m, 1H, H-3''), 7.62–7.52 (m, 1H, H-4''), 7.88–7.82 (m, 1H, H-5''), 7.73–7.65 (m, 1H, H-6''), 3.31–3.15 (m, 1H, CH), 1.38 (d, 6H, *J* = 6.9 Hz, CH_3_); ^13^C-NMR (CDCl_3_): 188.0, 166.0, 148.9, 145.8, 144.1, 141.5, 140.1, 133.4, 131.1, 130.5, 129.3, 125.2, 124.9, 34.4, 22.0; EA for C_16_H_15_N_3_O_3_ (297.31) calculated 64.64% C, 5.09% H, 14.13% N, found 64.71% C, 5.25% H, 14.15% N.

*(E)-1-(5-Isopropylpyrazin-2-yl)-3-(3-nitrophenyl)prop-2-en-1-one* (**4h**). Yellow solid; yield 43%; m.p. 178–180 °C; IR: 1673 (C=O), 1609 (C=C); ^1^H-NMR (CDCl_3_): 9.28 (d, 1H, *J* = 1.5 Hz, H-3'), 8.58 (d overlapped, 1H, *J* = 1.5 Hz, H-6'), 8.57 (t overlapped, 1H, J = 2.1 Hz, H-2''), 8.29 (d, 1H, *J* = 16.2 Hz, H-3), 8.26 (ddd overlapped, 1H, *J* = 1.1 and 2.1 and 8.1 Hz, H-6''), 8.01–7.96 (m, 1H, H-4''), 7.95 (d, 1H, *J* = 16.2 Hz, H-2), 7.62 (t, 1H, J = 8.1, H-5''), 3.32–3.16 (m, 1H, CH), 1.39 (d, 6H, *J* = 6.9 Hz, CH_3_); ^13^C-NMR (CDCl_3_): 188.2, 166.2, 148.7, 145.8, 144.0, 141.8, 141.6, 136.6, 134.6, 130.0, 124.8, 123.1, 122.8, 34.4, 22.0; EA for C_16_H_15_N_3_O_3_ (297.31) calculated 64.64% C, 5.09% H, 14.13% N, found 64.51% C, 5.26% H, 14.05% N.

*(E)-1-(5-Isopropylpyrazin-2-yl)-3-(4-nitrophenyl)prop-2-en-1-one* (**4i**). Yellow solid; yield 40%; m.p. 124–127 °C; IR: 1668 (C=O), 1609 (C=C); ^1^H-NMR (CDCl_3_): 9.28 (d, 1H, *J* = 1.4 Hz, H-3'), 8.57 (d, 1H, *J* = 1.4 Hz, H-6'), 8.30–6.25 (m, 2H, AA'BB', H-3'', H-5''), 8.29 (d, 1H, *J* = 16.0 Hz, H-3), 7.93 (d, 1H, *J* = 16.0 Hz, H-2), 7.89–7.82 (m, 2H, AA'BB', H-2'', H-6''), 3.32–3.15 (m, 1H, CH), 1.38 (d, 6H, *J* = 6.9 Hz, CH_3_); ^13^C-NMR (CDCl_3_): 188.6, 165.6, 158.5, 146.6, 145.4, 143.9, 141.5, 131.1, 127.7, 117.9, 116.0, 34.4, 22.0; EA for C_16_H_15_N_3_O_3_ (297.31) calculated 64.64% C, 5.09% H, 14.13% N, found 64.47% C, 5.10% H, 14.34% N.

*(E)-3-(4-Chlorophenyl)-1-(5-isopropylpyrazin-2-yl)prop-2-en-1-one* (**4j**). Yellow solid; yield 8%; m.p. 105–108 °C; IR: 1666 (C=O), 1608 (C=C); ^1^H-NMR (CDCl_3_): 9.27 (d, 1H, *J* = 1.4 Hz, H-3'), 8.55 (d, 1H, *J* = 1.4 Hz, H-6'), 8.15 (d, 1H, *J* = 15.9 Hz, H-3), 7.88 (d, 1H, *J* = 15.9 Hz, H-2), 7.68–7.60 (m, 2H, AA'BB', H-2'', H-6''), 7.43–6.35 (m, AA'BB', 2H, H-3'', H-5''), 3.30–3.14 (m, 1H, CH), 1.38 (d, 6H, *J* = 7.2 Hz, CH_3_); ^13^C-NMR (CDCl_3_): 188.4, 165.8, 146.1, 144.0, 143.6, 141.5, 136.7, 133.3, 130.0, 129.3, 120.8, 34.4, 22.0; EA for C_16_H_15_ClN_2_O (286.76) calculated 67.62% C, 5.27% H, 9.77% N, found 66.91% C, 5.36% H, 9.89% N.

### 3.2. Biological Evaluation

#### 3.2.1. Evaluation of* in Vitro* Antifungal Activity

The antifungal activity of all compounds was evaluated by the modified microdilution broth CSLI standards [50,51]. The organisms examined included *Candida albicans* ATCC 44859 (American Type Culture Collection, Manassas, VA, USA), *Candida tropicalis* 156, *Candida krusei* E 28, *Candida glabrata* 20/I, *Trichosporon asahii* 1188, *Aspergillus fumigatus* 231, *Lichtheimia corymbifera* (formerly *Absidia corymbifera*) 272, and *Trichophyton mentagrophytes* 445. All strains tested are clinical isolates obtained from the Department of Clinical Microbiology, University Hospital and Faculty of Medicine, Charles University, Prague, Czech Republic. Before testing each strain was subcultured on Sabouraud dextrose agar (SDA; Difco/Becton Dickinson, Detroit, MI, USA) and maintained on the same medium at 4 °C.

Fungal inocula were prepared by suspending yeasts, conidia, or sporangiospores in sterile 0.85% saline. The cell density was adjusted using a Bürker’s chamber to yield a stock suspension of 1.0 ± 0.2 × 10^5^ CFU/mL and 1.0 ± 0.2 × 10^6^ CFU/mL for yeasts and molds, respectively. The final inoculum was made by 1:20 dilution of the stock suspension with the test medium.

The compounds were dissolved in DMSO, and the antifungal activity was determined in RPMI 1640 media (KlinLab, Prague, Czech Republic) buffered to pH 7.0 with 0.165 M 3-morpholinopropane-1-sulfonic acid (Sigma-Aldrich, St. Louis, MO, USA). Controls consisted of medium and DMSO alone. The final concentration of DMSO in the test medium did not exceed 1% (v/v) of the total solution. The concentrations of the studied substances ranged from 500 to 0.488 μmol/L. The minimum inhibitory concentration (MIC), was defined as 80% or greater (for yeasts and yeast-like organisms—IC_80_), resp. 50% or greater (for molds—IC_50_) reduction of growth in comparison with the control. The values of MICs were determined after 24 and 48 h of static incubation at 35 °C. In the case of *T. mentagrophytes*, the MICs were recorded after 72 and 120 h due to its slow growth rate. Fluconazole, voriconazole and terbinafine were used as reference antifungal drug.

#### 3.2.2. Evaluation of* in Vitro* Antimycobacterial Activity

Primary screening of all compounds was conducted at 6.25 μg/mL against *Mycobacterium tuberculosis* H_37_Rv (ATCC 27294) in the BACTEC 12B medium using the Microplate Alamar Blue Assay (MABA). Compounds exhibiting fluorescence were tested in the BACTEC 460-radiometric system [52]. Compounds demonstrating at least 90% inhibition in the primary screen were re-tested at lower concentrations against *M. tuberculosis* H_37_Rv to determine the actual minimum inhibitory concentration (MIC) in the MABA. The MIC is defined as the lowest concentration effecting a reduction in fluorescence of 90% relative to controls.

The compounds that exhibited promising antimycobacterial activity were tested for cytotoxicity (CC_50_) in VERO cells at concentrations less than or equal to 10 times the MIC for *M. tuberculosis* H_37_Rv. After 72-h exposure, viability was assessed on the basis of cellular conversion of 1-(4,5-dimethylthiazol-2-yl)-2,5-diphenyltetrazolium (MTT) into a formazan product using the Promega CellTiter 96 Non-radioactive Cell Proliferation Assay. The selectivity index was then calculated as the ratio of the measured CC_50_ in VERO cells to the MIC described above.

## 4. Conclusions

A series of (*E*)-1-(5-isopropylpyrazin-2-yl)-3-phenylprop-2-en-1-ones with various substituents on the phenyl ring (ring B) was prepared and tested for antifungal and antimycobacterial activity. Their* in vitro* antifungal potency was compared to previously reported propyl analogs. Only *Trichophyton mentagrophytes* was susceptible to the tested compounds, and it was found that replacing a non-branched propyl with a branched isopropyl has no decisive and unequivocal influence on the* in vitro* antifungal activity against *T. mentagrophytes*. Unfortunately, no compound exhibited efficacy comparable with that of terbinafine, which is most widely used agent for treating mycoses caused by dermatophytes. In both biological assays, the highest* in vitro* potency was displayed by nitro- substituted derivatives. This confirms our previous findings about the positive effect of electron-withdrawing groups on the B-ring of chalcones on their antimicrobial activity.
